# Perception of asthma control among asthmatics seen inChest Clinic at Tertiary Hospital, Addis Ababa, Ethiopia

**DOI:** 10.1186/s12890-019-0959-7

**Published:** 2019-10-28

**Authors:** Tewodros H. Gebremariam, Charles B. Sherman, Neil W. Schluger

**Affiliations:** 1 Addis Ababa University, College of Heath Sciences, Lideta Sub-city Gambia St., P O Box 22787 code, 1000 Addis Ababa, Ethiopia; 2 Warren Alpert Medical School of Brown University, Providence, RI USA; 3 Division of Pulmonary, Allergy and Critical Care Medicine, Columbia, USA; 4 University College of Physicians and Surgeons, New York, NY USA

**Keywords:** GINA symptom assessment tool, Self-reported asthma control, SABA overuse

## Abstract

**Background:**

Patient awareness of asthma severity is important for optimal asthma management. However, there is often a discrepancy between physician assessment of asthma control based on guidelines and patient discernment of control. We compared physician and patient perception of asthma control in a clinic population seen at a tertiary hospital in Addis Ababa, Ethiopia.

**Methods:**

In this cross-sectional study, 182 consecutive patients with a physician diagnosis of asthma seen in Chest Clinic at Tikur Anbessa Specialized Hospital (TASH) between July and December 2015 were studied. Demographics, asthma symptoms, medication use in the past month, and self-perception of asthma control in the past 7 days were obtained from the clinic records. Physician assessed asthma control was based on the GINA asthma symptom control assessment tool. Lung function was measured using a Diagnostic EasyOne Plus model 2001 SN spirometer. The institutional review board approved the study protocol.

**Results:**

Of the 182 subjects, 68.1% were female. The mean age was 52 ± 12 years, and the mean (SD) duration of asthma was 19.4 ± 12.7 years. Forty-four (24.2%) patients had physician determined well-controlled asthma and 138 (75.8%) patients had physician determined partly controlled/uncontrolled asthma. One hundred and fifty-one (83%) patients thought their asthma control was good. However, the degree of concordance between physician evaluation and patient perception of asthma control was low (kappa index = 0.09). On multivariate analysis, self-perceived poor asthma control was associated with any activity limitation due to asthma and inconsistent inhaled corticosteroid use.

**Conclusion:**

In our study, the first of its kind in Ethiopia, a high percent of patients with physician determined well-controlled asthma has appropriate perception of their disease state. However, those patients with partly controlled/uncontrolled asthma had poor self-perception of their disease, emphasizing the need for further patient education. These conclusions may be especially useful in the care of asthmatics from other low-income countries.

## Background

Physicians often rely on guidelines to determine asthma control. According to the Global Initiative for Asthma (GINA), asthma control is defined as the degree to which treatment has reduced the symptoms of asthma, and prevented disease exacerbations, worsening lung function, and drug side effects [1]. The three levels of asthma control as recommended by GINA include: well-controlled, partially controlled, and uncontrolled [1]. The presence or absence of typical asthma symptoms (breathlessness, chest tightness, wheezing, cough) more than two times per week, nocturnal awakening, use of short acting beta agonist (SABA) more than two times per week, and activity limitation are the basis for this classification [1].

Appropriate asthma medication use is essential for disease management and often depends on patient self-perception of asthma control. Unfortunately, patients often believe their asthma to be well-controlled despite objective evidence of significant impairment [2–4]. The Asthma Insights and Reality surveys, a worldwide assessment of severity and control of asthma in children and adults, found that many patients consider their asthma to be controlled despite experiencing severe symptoms [5]. In another survey done in Europe and Canada, 81% of study participants thought their asthma was well or completely controlled, yet 26% reported symptoms daily or on most days over the past 4 weeks [6].

The interplay between physician and patient perception of asthma control remains an important determinant of asthma management [7–9]. Inaccurate perception of asthma severity on either part may negatively influence asthma prognosis [10]. This study, the first of its kind in Ethiopia, was designed to determine the correlation between physician assessed asthma control using the GINA asthma symptom control tool and patient self-perception of disease state.

## Methods

The study was conducted in the chest clinic of Tikur Anbessa Specialized Hospital (TASH) in Addis Ababa. TASH has 850 beds and serves approximately 400,000 patients per year, making it the largest tertiary hospital in Ethiopia. The chest clinic at TASH sees a large number of asthma patients monthly, over 150visits/month, and has a well-organized and robust longitudinal database. Medical residents, pulmonary and critical care medicine fellows, and faculty members provide care in the clinic, which is open three half-days per week. Pharmacists both provide medication and instruction on proper inhaler technique.

The study used a cross-sectional design. Between July and December 2015, all consecutive physician-diagnosed asthmatics ≥18 years of age were included if they had used asthma medication for the last 6 months or more. Patients were excluded from the study if there was a suspicion of bronchiectasis, active lung infection, chronic obstructive pulmonary disease (COPD), or if their chart had incomplete data. Patient charts were reviewed for modifiable risk factors for poor asthma control (such as smoking, biomass fuel exposures, etc.) and co-morbidities. In addition, respiratory symptoms (i.e., wheeze, breathlessness, cough, and chest tightness), current medications, and demographics were recorded. Patient’s perception of their asthma control over the past week was also obtained. Patient-reported worsening of respiratory symptoms for over 48 h in the past 12 months was defined as an asthma exacerbation. Other definitions included allergic rhinitis, if patient report recurrent rhinorrhea and sneezing unrelated to symptoms of upper respiratory tract infections and GERD, if patient had heartburn requiring H2 blocker or proton pump inhibitor.

A regularly calibrated Diagnostic EasyOne Plus model 2001 SN spirometer was used to measure forced expiratory volume in the first second (FEV1), forced vital capacity(FVC) and FEV1/FVC ratio. The criteria established by the European Respiratory Society and the American Thoracic Societyfor acceptability and reproducibility of testing were used [11].

GINA asthma symptom control assessment tool, which was determined by assessing frequency of symptoms, nighttime awakening, activity limitation, and frequency of rescue inhaler use, was used to determine physician assessment of asthma control [1]. The tool matches well with other standardized asthma control scores [12, 13]. Total absence of nighttime symptoms, complete lack of activity limitations, rescue inhalers used no more than twice a week and daytime symptoms occurring no more than twice a week defined well-controlled asthma. Uncontrolled asthma was reported if any three or more of these individual features were present within any week and partially-controlled asthma if the patient’s symptoms did not fall into either well-controlled or uncontrolled asthma definitions [1]. For the purposes of analysis, patients were grouped into well-controlled asthmatics and partially- or poorly-controlled asthmatics.

IBM SPSS statistics Version 20 (Armonk, NY: IBM Corp) was used to perform the statistical analyses. Mean, median, standard deviation, or interquartile ranges were used to describe continuous data, while categorical variables were presented as frequencies and percentages. The Student t-test was utilized to compare continuous variables. Categorical variables were compared with the Chi-square test and the Fisher exact test for independent samples.

For the analyses, first, the degree of patient-physician concordance regarding the perception of asthma control was determined using the Kappa index. Then, the frequency of patients with poor perception of asthma control was determined. Patients were divided into two groups, according to their perception of asthma control in a subsequent comparative analysis using univariate logistic regression. Patients that agreed with the physician’s perception of asthma control (i.e., patients with good perception) were classified as group one and those who did not agree with the physician’s perception of asthma control (i.e., patients with poor perception) was classified as group two. Finally, independent factors associated with poor asthma control perception were identified using multivariate logistic regression models, using a stepwise strategy of variables that showed a *p* ≤ 0.10. Odds ratios (OR) and their 95% confidence intervals (CI) were determined and a *p* < 0.05 was considered as statistically significant.

In summary, this study shared similar methodology including study setting, design, participants and data collection from a previously published work [14]. The institutional review board approved the study protocol and all subjects signed informed consent.

## Results

### Socio-demographic and clinical characteristics of patients

One hundred eighty-eight subjects were recruited, with 6 subjects having incomplete data, leaving 182 for analysis. Of these 182 study subjects, 68.1% were female. The mean (SD) age of the study subjects was 52 ± 12 years with a mean (SD) duration of asthma at 19.4 ± 12.7 years. Only thirteen (7.3%) subjects had a history of tobacco use. The vast majority of patients lived in urban centers-165 (90.6%) of 182. Nearly one-third (*n* = 66 or 37.7%) used biomass fuel for cooking. Illiteracy was seen in fifty (27.4%) patients with inability to read or write. Only 13.6% (9 of 66 of subjects with recorded weights) had body mass index (BMI) ≥ 30.

Reported asthma symptoms are described in Table 1. Nearly half of patients had an asthma exacerbation in the past 12 months (93 subjects or 51.1%). Commonly used asthma medication use is reported in Table 2. Fifty-eight (31%) subjects reported not using controller medications, and of those using inhalers, improper inhaler technique was observed in 62 (34.6%) subjects. Patients carrying an asthma diagnosis for over 5 years had a tendency to report worse asthma control, but statistical significance was not reached (OR 0.44(0.17,1.12) *p* = 0.08).

**Table 1:** Reported asthma symptoms in 182 patients seen in Chest Clinic at Tikur Anbessa Specialized Hospital

Symptom	Frequency	Percent
Nighttime awakening due to asthma	117	64.3
Daytime symptoms (wheeze or dyspnea) more than twice per week	105	57.7
Activity limitations due to asthma	52	28.6

**Table 2:** Asthma medication use in182 subjects seen in Chest Clinic at Tikur Anbessa Specialized Hospital

Type of medication	Frequency	Percent
Short acting beta agonist (SABA)	173	95.1
Inhaled corticosteroids (ICS)	105	57.7
Consistent ICS use	95	52.2
Combination ICS and long acting beta agonist (LABA)	19	10.4
Oral steroids	25	13.7

Spirometry was performed in 96 (52.7%) subjects. In 73 of the 96 (96%) studied, FEV1/FVC was < 70%. FEV1 was < 60% in 43 (58.9%), 60–80% in 29 (39.7%), and > 80% in 1 (1.4%).

### Physician patient disease control perception- concordance/discordance

Forty-four (24.2%) patients had well-controlled asthma and 138 (75.8%) patients had physician determined partly controlled/uncontrolled asthma. Asthma control was perceived as good in 151 (83%) patients and as poor in 31 (17%) subjects (Table 3). The degree of concordance between physician and patient perception of asthma control was low (kappa index = 0.09) (*p* < 0.03). Forty-two (95.5%) patients among the well-controlled asthma group thought their asthma control was good. However, only 29(21%) patients with partly controlled/uncontrolled asthma thought their asthma control was poor.

**Table 3:** Frequency of patients with poor perception of asthma control

		Asthma control by Physician assessment (based on GINA)	
		Well Controlled (*n* = 44)	Partly controlled/uncontrolled (*n* = 138)
Patient Perception of asthma	Good Control	42 (95.5%)	109 (79%)
	Poor Control	2 (4.5%)	29 (21%)

*GINA* Global Initiative for Asthma management and prevention

Tables 4 and 5 respectively show the demographic and clinical characteristics of the patients grouped as self-reported good asthma control or self-perceived poor control. On bivariate analysis, night awakening, any activity limitation due to asthma, SABA reliever use >2x/week, overall poor GINA control, incorrect inhaler use, and inconsistent ICS use were found to be associated with self-report poor asthma control. On multivariate analysis, only any activity limitation due to asthma and inconsistent ICS use were associated with self-perceived poor asthma control.

**Table 4:** Demographic characteristics of patients grouped as self-reported good asthma control or self-perceived poor control

Variable		Self-reported good asthma control (*n* = 151)	Self-perceived poor asthma control (*n* = 31)	*P* value	Adjusted *P* value	OR
Age(yr), Mean(SD)		51.6(12.4)	52.3(10.55)	0.76		0.31(0.05–4.03)
Female(%)		106(70.2)	18(58.1)	0.19		0.59(0.27–1.30)
Duration of Asthma (yr), Mean(SD)		19.6(11.6)	18.2(17.5)	0.57		0.57(0.35–6.42)
Level of education (%)	Illiterate	37(24.5)	12(38.7)	0.11		1.95(0.86–4.38)
	Literate	114(75.5)	19(61.3)			
Smoking	Yes	15(9.9)	3(9.7)	0.97		0.97 (0.26–3.58)
	No	136(90.1)	28(90.3)			
Biomass use for cooking	Yes	51(34.9)	15(51.7)	0.09	0.34	0.62 (0.23–1.65)
	No	95(65.1)	14(48.3)

**Table 5:** Clinical characteristics of patients grouped as self-reported good asthma control or self-perceived poor control

Variable		Self-reported good asthma control (*n* = 151)	Self-perceived poor asthma control (*n* = 31)	*P* value	Adjusted *P* value	OR
Day time symptom(>2x/wee)	Yes	74(49)	31(100)	0.19		1.73(0.69–3.08)
	No	77(51)	0(0)			
Night awakening	Yes	88(58.3)	30(96.8)	0.003	0.13	5.499(0.59–51.13)
	No	63(41.7)	1(3.2)			
Use of reliever /SABA >2x/week	Yes	87(57.6)	28(90.3)	0.002	0.076	4.29(0.86–21.45)
	No	64(42.4)	3(9.7)			
Any Activity limitation	Yes	58(38.4)	24(77.4)	0.000	0.045	3.05 (1.03–9.07)
	No	93(61.6)	7 (22.6)			
Overall GINA control	Well controlled	80(53)	5(16.1)	0.001	0.87	0.89 (0.21–3.66)
	Partly controlled/Uncontrolled	71(47)	26(83.9)			
Exacerbation	Yes	74(49)	19(61.3)	0.22		1.65 (0.75–3.63)
	No	77(51)	12 (38.7)			
Incorrect Inhaler Technique	Yes	46(30.9)	16(53.3)	0.02	0.67	1.23 (0.48–3.12)
	No	103(69.1)	14(46.7)			
Consistent ICS use	Yes	85(56.3)	10(32.3)	0.002	0.02	4.63 (1.73–12.42)
	No	66(43.7)	21(67.7)			
FVC	≥ 80	44(55.7)	11(64.7)	0.497		0.69 (0.23–2.04)
	< 80	35(44.3)	6(35.3)			
FEV1	≥ 80	19(24.1)	5(29.4)	0.64		0.76 (0.24–2.43)
	< 80	60(75.9)	12(70.6)			

*SABA* Short acting beta agonist, *GINA* Global Initiative for Asthma management and prevention, *ICS* Inhaled corticosteroid, *FVC* Forced vital capacity, *FEV1* Forced Expiratory volume in the first second

### Discussion

In this study of asthma control perception performed in an outpatient referral clinic, a substantial proportion of asthmatics had poor self-recognition of disease severity. Additionally, the degree of concordance between physician assessment, as evaluated by the GINA asthma symptom control assessment tool, and patient perception of asthma control was low. Most patients (79%) perceived their asthma to be well-controlled whereas in actuality, it was partially controlled or uncontrolled by physician assessment. In a similar study done in Portugal, over half of the patients that perceived their asthma as being well-controlled actually had poor control [15].

Underestimation of asthma control can result in an increased risk of hospitalization and morbidity due to postponement of treatment [16]. Furthermore, poor perception of asthma symptoms has been shown to correlate with asthma exacerbations and its complications [17, 18].

Several studies have shown that some asthmatics have poor recognition of increasing airflow obstruction. Reck et al. found that 21 of 53 (39%) mild asthmatics were unable to identify methacholine-induced bronchoconstriction [19]. In another study from Brazil, 51 % of patients did not accurately characterize their degree of airway obstruction when using a visual analog symptom scale compared to spirometric data [20]. Disparities between patients’ perception of asthma control and actual control have been observed in several other studies [2–6].

Accurate reporting of patient symptoms is imperative for good asthma management. In the COPD and Asthma Detection, Intervention, and Monitoring Program, 66 % of asthmatics did not spontaneously report symptoms to their physicians [21]. Consequently, physicians prescribed an inadequate treatment regimen based on underestimation of disease severity. In contrast, some patients may overestimate their asthma symptoms, also adversely influencing management. Therefore, patient perception of asthma control, primarily based on reported symptoms, can have significant influence on the formulation and execution of appropriate asthma treatment.

Any activity limitation due to asthma was one of the factors associated with self-report of poor asthma control. Other components of the GINA asthma symptom control assessment tool such as night awakening, SABA reliever use >2X/week and overall GINA score were found to be significant on bivariate analysis and failed to attain significance in the multivariate analysis. As opposed to the other items in GINA tool, activity limitation may be the one that is better perceived by the patients. Haselkom and associates has similarly demonstrated that patients with uncontrolled asthma are at higher risk for any activity limitation as compared to those with controlled asthma [22].

In our study, the other factor that was associated with self-report of poor asthma control was inconsistent ICS use. Known benefits of ICS use in the management of asthma include: reduction in asthma symptoms, improved lung function, better quality of life, fewer exacerbations, and less asthma-related hospitalizations or death [23–25]. Therefore, it makes sense that those asthmatics with poor asthma control would also have inconsistent use of ICS.

A previous study done in high-income country found a low rate of patient-physician concordance when elderly patients were asked about respiratory symptoms, especially if chronic in nature [26]. The average age for this study was 69.6 years (range 55 to 94) which was older than our study group, which was 52 years. However, they too found that in more than half of the patients, asthma perception was discordant between patients and physicians.

Our overall findings are similar to those of many other studies [27–29] done in high-income countries. In these studies, as in our own, patients often tolerated respiratory symptoms and poor asthma control as part of living with their disease. Our results are the first from Ethiopia and may be especially useful in the care of asthmatics from other low-income countries.

Several studies have demonstrated that patient education can lead to improved asthma control [30, 31]. Although performed in high income countries, we believe that we can adapt many of their findings to our own setup. Targeted education in the clinic by resident physicians, senior pulmonologists, pharmacists, and nurses can help correct the misperception that most of our patients have that their symptoms and limitations are to be expected. In turn, this should lessen the discordance found and greatly improve asthma control for our clinic patients. As most of our patients are unable to read or write, we don’t think that written literature will be as effective as suggested for those living in high-income countries.

## Limitations

This study has several limitations. Patients were recruited from the chest clinic of a large tertiary.

public hospital, possibly influencing generalizability of the findings. The time of the study selected, between July and December, which are rainy and cold (winter and spring) in Ethiopia, might have contributed to the high level of poor asthma control (79% of poor control). Physician diagnosis was used to identify study participants, making misclassification possible. Bronchodilator responsiveness was not obtained and therefore some of the study patients may have had COPD rather than asthma. In addition, we did not assess medication use for concomitant diseases (i.e., rhinitis, conjunctivitis or GERD)**,** which may have underestimated the level of asthma control in our study. Treatment adherence was not regularly assessed, which could have contributed to the high percent of poor asthma control. Finally, we did not assess misuse or lack of use of inhaler medications, which may have led to the perception of good control by patients but poor control by physicians using the GINA asthma symptom control assessment tool.

## Conclusion

In our study, the first of its kind in Ethiopia, a high percent of patients with physician determined well-controlled asthma had appropriate awareness of their disease state. However, those patients with partly controlled/uncontrolled asthma had poor self-perception of their disease, highlighting the need for further patient education. Asthma education can help patients identify disease symptoms, adhere to treatment plans, avoid environmental triggers, and seek treatment when symptoms worsen. We believe this will improve asthma control in our patients*.* These conclusions may be especially useful in the care of asthmatics from other low-income countries.

## Data Availability

All data generated or analyzed during this study are included in this published article.

## Abbreviations

AAU CHS: Addis Ababa University College of Health Sciences
BMI: Body Mass Index
COPD: Chronic Obstructive Pulmonary Disease
FEV1: Forced Expiratory volume in the first second.
FVC: Forced vital capacity
GINA: Global Initiative for Asthma management and prevention
ICS: Inhaled corticosteroid
LABA: Long acting beta agonist
SABA: Short acting beta agonist
TASH: Tikur Anbessa Specialized Hospital
